# Lithio­marsturite, LiCa_2_Mn_2_Si_5_O_14_(OH)

**DOI:** 10.1107/S1600536811047581

**Published:** 2011-11-16

**Authors:** Hexiong Yang, Robert T. Downs, Yongbo W. Yang

**Affiliations:** a Department of Geosciences, University of Arizona, 1040 E. 4th Street, Tucson, Arizona 85721-0077, USA; b Department of Chemsitry and Biochemistry, University of Arizona, 1306 E. University Blvd, Tucson, Arizona 85721-0041, USA

## Abstract

Lithio­marsturite, ideally LiCa_2_Mn_2_Si_5_O_14_(OH), is a member of the pectolite–pyroxene series of pyroxenoids (hydro­pyroxenoids) and belongs to the rhodonite group. A previous structure determination of this mineral based on triclinic symmetry in space group *P* 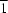 by Peacor *et al.* [*Am. Mineral*. (1990), **75**, 409–414] converged with *R* = 0.18 without reporting any information on atomic coordinates and displacement param­eters. The current study redetermines its structure from a natural specimen from the type locality (Foote mine, North Carolina) based on single-crystal X-ray diffraction data. The crystal structure of lithio­marsturite is characterized by ribbons of edge-sharing CaO_6_ and two types of MnO_6_ octa­hedra as well as chains of corner-sharing SiO_4_ tetra­hedra, both extending along [110]. The octa­hedral ribbons are inter­connected by the rather irregular CaO_8_ and LiO_6_ polyhedra through sharing corners and edges, forming layers parallel to (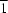
1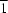), which are linked together by the silicate chains. Whereas the coordination environments of the Mn and Li cations can be compared to those of the corresponding cations in nambulite, the bonding situations of the Ca cations are more similar to those in babingtonite. In contrast to the hydrogen-bonding scheme in babingtonite, which has one O atom as the hydrogen-bond donor and a second O atom as the hydrogen-bond acceptor, our study shows that the situation is reversed in lithio­marsturite for the same two O atoms, as a consequence of the differences in the bonding environments around O atoms in the two minerals.

## Related literature

For a previous structural study of lithiomartusite, see: Peacor *et al.* (1990 ▶). For other minerals of the rhodonite group, see: Liebau *et al.* (1959 ▶); Peacor & Niizeki (1963 ▶); Araki & Zoltai (1972 ▶); Kosoi (1976 ▶); Narita *et al.* (1975 ▶); Tagai *et al.* (1990 ▶); Orlandi *et al.* (1998 ▶); Armbruster (2000 ▶). For the definition of polyhedral distortion, see: Robinson *et al.* (1971 ▶). For bond-valence calculations, see: Brese & O’Keeffe (1991 ▶).

## Experimental

### 

#### Crystal data

LiCa_2_Mn_2_Si_5_O_14_(OH)
*M* *_r_* = 578.44
Triclinic, 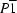

*a* = 7.6467 (3) Å
*b* = 11.7315 (6) Å
*c* = 6.8100 (3) Å
α = 91.874 (4)°
β = 94.465 (3)°
γ = 105.933 (3)°
*V* = 584.72 (5) Å^3^
*Z* = 2
Mo *K*α radiation
μ = 3.65 mm^−1^
*T* = 293 K
0.06 × 0.05 × 0.05 mm

#### Data collection

Bruker APEXII CCD area-detector diffractometer
Absorption correction: multi-scan (*SADABS*; Sheldrick 2005 ▶) *T*_min_ = 0.811, *T*_max_ = 0.839
14045 measured reflections
4191 independent reflections
3317 reflections with *I* > 2σ(*I*)
*R*_int_ = 0.035

#### Refinement

*R*[*F* ^2^ > 2σ(*F* ^2^)] = 0.033
*wR*(*F* ^2^) = 0.078
*S* = 1.03
4191 reflections
230 parameters
Only H-atom coordinates refined
Δρ_max_ = 1.26 e Å^−3^
Δρ_min_ = −0.56 e Å^−3^

### 

Data collection: *APEX2* (Bruker, 2004 ▶); cell refinement: *SAINT* (Bruker, 2004 ▶); data reduction: *SAINT*; program(s) used to solve structure: *SHELXS97* (Sheldrick, 2008 ▶); program(s) used to refine structure: *SHELXL97* (Sheldrick, 2008 ▶); molecular graphics: *XtalDraw* (Downs & Hall-Wallace, 2003 ▶); software used to prepare material for publication: *publCIF* (Westrip, 2010 ▶).

## Supplementary Material

Crystal structure: contains datablock(s) I, global. DOI: 10.1107/S1600536811047581/ru2015sup1.cif

Structure factors: contains datablock(s) I. DOI: 10.1107/S1600536811047581/ru2015Isup2.hkl

Additional supplementary materials:  crystallographic information; 3D view; checkCIF report

## Figures and Tables

**Table 1:** Hydrogen-bond geometry (Å, °)

*D*—H⋯*A*	*D*—H	H⋯*A*	*D*⋯*A*	*D*—H⋯*A*
O11—H1⋯O1^i^	1.07 (4)	1.44 (4)	2.462 (3)	157 (3)

Symmetry code: (i) 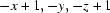.

**Fig. 1.**
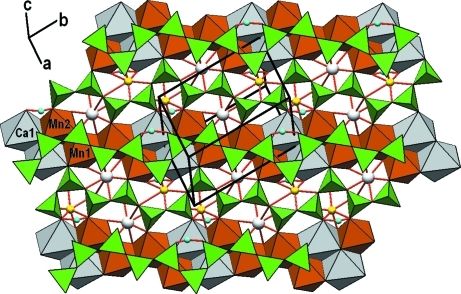
Crystal structure of lithiomarsturite. The green tetrahedra represent SiO4 groups. Large gray, medium yellow, and small bright blue sphares represent Ca2, Li1, H1 atoms, respectively. Both ribbons of edge-sharing octahedra and chains of vertex-sharing tetrahedra run parallel to [110].

## supplementary crystallographic information

### Comment

Lithiomarsturite, ideally LiCa_2_Mn_2_Si_5_O_14_(OH), is a member of the pectolite-pyroxene series of pyroxenoids (hydropyroxenoids) and belongs to the rhodonite group, which includes rhodonite MnSiO_3_, babingtonite Ca_2_Fe^2+^Fe^3+^Si_5_O_14_(OH), manganbabingtonite Ca_2_Mn^2+^Fe^3+^Si_5_O_14_(OH), scandiobabingtonite (Ca,Na)_2_(Fe^2+^,Mn)(Sc,Fe^3+^)Si_5_O_14_(OH), nambulite LiMn_4_Si_5_O_14_(OH), natronambulite NaMn_4_Si_5_O_14_(OH), marsturite NaCa_2_Mn_2_Si_5_O_14_(OH), and lithiomarsturite. Thus far, only the crystal structures of four minerals in this group have been determined, including rhodonite (Liebau *et al.* 1959; Peacor & Niizeki, 1963), babingtonite (Araki & Zoltai, 1972; Kosoi, 1976), nambulite (Narita *et al.*, 1975), and scandiobabingtonite (Orlandi *et al.*, 1998).

Lithiomarsturite was first described by Peacor *et al.* (1990) from the Foote mine in North Carolina, with space group *P*1, and unit-cell parameters *a* = 7.652 (3), *b* = 12.119 (3), *c* = 6.805 (2) Å, α = 85.41 (2), β = 94.42 (3), γ = 111.51 (2)°. The reported empirical chemical formula, derived from electron and ion microprobe analyses, is Li_1.01_Ca_1.98_Mn_1.35_Fe_0.56_Mg_0.10_H_1.00_Si_5.00_O_15_, which can be simplified as LiCa_2_Mn_2_Si_5_O_14_(OH). Although Peacor *et al.* (1990) showed that this mineral belongs to the rhodonite group, they did not provide any detailed information on atomic coordinates and displacement parameters, because their structure refinement with 2338 single-crystal X-ray diffraction intensity data converged with *R* = 18%, due probably to the presence of common chain-periodicity faults. Since then, no further study on lithiomarsturite has been reported. This study presents the first structure refinement of lithiomarsturite based on single-crystal X-ray diffraction data.

Lithiomarsturite is isotypic with nambulite. Its structure is characterized by ribbons of edge-sharing Ca1O_6_, Mn1O_6_ and Mn2O_6_ octahedra and chains of corner-sharing SiO_4_ tetrahedra, both extending along [110] (Fig. 1). The octahedral ribbons are interconnected by the Ca2O_8_ and Li1O_6_ polyhedra through sharing corners and edges to form layers parallel to (1 1 1), which are linked together by the silicate chains. Whereas the Ca1, Mn1, and Mn2 octahedra are fairly regular, the Ca2O_8_ and Li1O_6_ polyhedra are rather irregular. The average Mn1—O and Mn2—O bond distances are 2.202 and 2.172 Å, respectively, both of which are slightly shorter than the corresponding Mn—O distances (2.233 and 2.188 Å) in nambulite (Narita *et al.*, 1975). Within 2.9 Å, Li is coordinated to six O atoms in lithiomarsturite (the next nearest O atom is at 3.12 Å), with an average Li—O distance of 2.322 Å, but to eight O atoms in nambulite, with an average Li—O distance of 2.485 Å (Narita *et al.*, 1975). This difference can be ascribed mostly to the replacement of the significant amount of Li (43%) by Na in nambulite examined by Narita *et al.* (1975).

The two Ca polyhedra in lithiomarsturite are better compared to those in babingtonite (Tagai *et al.*, 1990; Armbruster, 2000). The Ca1O_6_ octahedra in lithiomarsturite, on the one hand, are much less distorted than those in babingtonite in terms of both the octahedral angle variance (OAV) and quadratic elongation (OQE) (Robinson *et al.*, 1971), which are 146 and 1.040, respectively, for the former, and 311 and 1.092 for the latter. The greater distortion of the Ca1 octahedra in babingtonite is understandable, because they share edges with the Fe1O_6_ and Fe2O_6_ octahedra in the octahedral ribbons, which are primarily occupied by cations with different sizes and charges (Fe^2+^ and Fe^3+^, respectively). In contrast, both Mn1O_6_ and Mn2O_6_ octahedra that share edges with the Ca1O_6_ octahedra in lithiomarsturite (Fig. 1) are filled with the same kind of cation (Mn^2+^). On the other hand, the Ca2 cations in lithiomarsturite and babingtonite exhibit similar coordination environments, both of which have seven Ca—O bond lengths shorter than 2.65 Å and one Ca—O bond length greater than 2.84 Å. If we only take the seven shorter Ca—O bond lengths into account, then a calculation of the bond-valence sums using the parameters given by Brese & O'Keeffe (1991) yields 2.03 and 2.09 v.u. for the Ca2 cations in lithiomarsturite and babingtonite, respectively, suggesting that the Ca2 cations may be only bonded to seven, rather eight, O atoms in these two minerals. At least, this should be the case for Ca2 in lithiomarsturite, because the next nearest O atom (except the seven closer O atoms) is 3.059 Å away.

Very intriguingly, both previous neutron and X-ray diffraction studies (Tagai *et al.*, 1990; Armbruster, 2000) have demonstrated that the hydrogen bonding in babingtonite is between O1 and O11, with the former as the H-donor and the latter as the H-acceptor. However, our study shows the opposite case for lithiomarsturite, in which O11 is the H-donor and O1 the H-acceptor. This difference is the direct result of the change in the bonding environments around O1 and O11 in the two minerals. In babingtonite, both O1 and O11 are bonded to two non-hydrogen cations, with O1 to Si1 and Ca1, and O11 to Si4 and Fe2, but in lithiomarsturite, O1 is bonded to three non-hydrogen cations (Si1, Ca1, and Li1), and O11 to two (Si4 and Mn2). Note that Fe2 in babingtonite is chiefly trivalent Fe^3+^, whereas Mn2 in lithiomarsturite is essentially divalent Mn^2+^. As a consequence of this coupled effect, the more underbonded O1 (relative to O11) in babingtonite becomes less underbonded in lithiomarsturite. Because the more underbonded O atom will be more tightly bonded to the H atom to better satisfy its bond-valence requirement, we see the change from O1 being the H-donor in babingtonite to O11 in lithiomarsturite. Accordingly, it is most likely that other members of the rhodonite group that contain Li or Na as an essential component, such as nambulite, natronambulite, and marsturite, may all behave as lithiomarsturite in terms of the hydrogen bonding scheme, with O11 being the H-donor and O1 the H-acceptor. For some specific chemical compositions, nevertheless, it may also be possible that the H atom is situated halfway between O1 and O11 or hops between the two positions.

### Experimental

The lithiomarsturite sample used in this study is from the type locality: the Foote mine, Kings Mountains, North Carolina, USA, and is in the collection of the RRUFF project (deposition No. R100094; http://rruff.info). The chemical composition analyzed by Peacor *et al.* (1990) was adopted for the structure refinement. To keep consistent with the unit-cell settings for other minerals in the rhodonite group, such as rhodonite, babingtonite, and nambulite, we have adopted a unit-cell setting that differs from the one given by Peacor *et al.* (1990). The matrix for the transformation from the unit-cell setting of Peacor *et al.* (1990) to ours is [-1 0 0 / 1 1 0 / 0 0 - 1]. The labeling scheme of the atoms in lithiomarsturite is similar to that for numbulite (Narita *et al.*, 1975).

### Refinement

The H atom was located from difference Fourier syntheses and its position refined freely with a fixed isotropic displacement (*U_iso_* = 0.04). During the structure refinements, Fe was treated as Mn, because of their similar X-ray scattering powers. The final refinement assumed an ideal chemistry for lithiomarsturite, as the overall effects of the trace amount of Mg on the final structure results are negligible. The highest residual peak in the difference Fourier maps was located at (0.5254, 0.8418, 0.2798), 1.48 Å from O13, and the deepest hole at (0.1839, 0.5391, 0.3061), 0.49 Å from Ca2.

### Crystal data

**Table:**

LiCa_2_Mn_2_Si_5_O_14_(OH)	*Z* = 2
*M**_r_* = 578.44	*F*(000) = 568
Triclinic, *P*1	*D*_x_ = 3.285 Mg m^−^^3^
Hall symbol: -P 1	Mo *K*α radiation, λ = 0.71073 Å
*a* = 7.6467 (3) Å	Cell parameters from 2086 reflections
*b* = 11.7315 (6) Å	θ = 3.5–28°
*c* = 6.8100 (3) Å	µ = 3.65 mm^−^^1^
α = 91.874 (4)°	*T* = 293 K
β = 94.465 (3)°	Cube, light gray
γ = 105.933 (3)°	0.06 × 0.05 × 0.05 mm
*V* = 584.72 (5) Å^3^	

### Data collection

**Table:**

Bruker APEXII CCD area-detector diffractometer	4191 independent reflections
Radiation source: fine-focus sealed tube	3317 reflections with *I* > 2σ(*I*)
graphite	*R*_int_ = 0.035
φ and ω scan	θ_max_ = 32.6°, θ_min_ = 3.0°
Absorption correction: multi-scan (*SADABS*; Sheldrick 2005)	*h* = −11→11
*T*_min_ = 0.811, *T*_max_ = 0.839	*k* = −17→17
14045 measured reflections	*l* = −10→10

### Refinement

**Table:**

Refinement on *F*^2^	Secondary atom site location: difference Fourier map
Least-squares matrix: full	Hydrogen site location: difference Fourier map
*R*[*F*^2^ > 2σ(*F*^2^)] = 0.033	Only H-atom coordinates refined
*wR*(*F*^2^) = 0.078	*w* = 1/[σ^2^(*F*_o_^2^) + (0.0354*P*)^2^ + 0.2944*P*] where *P* = (*F*_o_^2^ + 2*F*_c_^2^)/3
*S* = 1.03	(Δ/σ)_max_ = 0.006
4191 reflections	Δρ_max_ = 1.26 e Å^−^^3^
230 parameters	Δρ_min_ = −0.56 e Å^−^^3^
0 restraints	Extinction correction: *SHELXL97* (Sheldrick, 2008), Fc^*^=kFc[1+0.001xFc^2^λ^3^/sin(2θ)]^-1/4^
Primary atom site location: structure-invariant direct methods	Extinction coefficient: 0.0023 (5)

### Special details

**Table:**

Geometry. All e.s.d.'s (except the e.s.d. in the dihedral angle between two l.s. planes) are estimated using the full covariance matrix. The cell e.s.d.'s are taken into account individually in the estimation of e.s.d.'s in distances, angles and torsion angles; correlations between e.s.d.'s in cell parameters are only used when they are defined by crystal symmetry. An approximate (isotropic) treatment of cell e.s.d.'s is used for estimating e.s.d.'s involving l.s. planes.
Refinement. Refinement of *F*^2^ against ALL reflections. The weighted *R*-factor *wR* and goodness of fit *S* are based on *F*^2^, conventional *R*-factors *R* are based on *F*, with *F* set to zero for negative *F*^2^. The threshold expression of *F*^2^ > σ(*F*^2^) is used only for calculating *R*-factors(gt) *etc*. and is not relevant to the choice of reflections for refinement. *R*-factors based on *F*^2^ are statistically about twice as large as those based on *F*, and *R*- factors based on ALL data will be even larger.

### Fractional atomic coordinates and isotropic or equivalent isotropic displacement parameters (Å^2^)

**Table:**

	*x*	*y*	*z*	*U*_iso_*/*U*_eq_	
Li1	0.6570 (12)	0.1276 (10)	0.3338 (12)	0.067 (3)	
Ca1	0.80601 (7)	0.94132 (4)	0.13944 (7)	0.01028 (10)	
Ca2	0.23393 (7)	0.52331 (5)	0.28673 (7)	0.01254 (10)	
Mn1	0.59386 (5)	0.64710 (3)	0.06116 (5)	0.00721 (8)	
Mn2	0.04394 (5)	0.23677 (3)	0.18152 (5)	0.00722 (8)	
Si1	0.28154 (9)	0.05291 (6)	0.34656 (9)	0.00840 (13)	
Si2	0.46371 (9)	0.32017 (6)	0.42345 (9)	0.00704 (12)	
Si3	0.80739 (9)	0.44983 (6)	0.21522 (9)	0.00627 (12)	
Si4	0.99738 (9)	0.71828 (6)	0.30096 (9)	0.00756 (12)	
Si5	0.33975 (9)	0.84444 (6)	0.10838 (9)	0.00758 (12)	
O1	0.2071 (3)	−0.00760 (16)	0.5439 (3)	0.0155 (4)	
O2	0.1231 (2)	0.06860 (15)	0.1893 (3)	0.0116 (3)	
O3	0.4399 (2)	0.17592 (14)	0.4144 (2)	0.0104 (3)	
O4	0.3253 (2)	0.34697 (15)	0.2519 (2)	0.0108 (3)	
O5	0.5434 (2)	0.62430 (15)	0.3623 (2)	0.0113 (3)	
O6	0.6815 (2)	0.37564 (15)	0.3790 (2)	0.0107 (3)	
O7	0.9617 (2)	0.39040 (15)	0.1616 (3)	0.0116 (3)	
O8	0.6769 (2)	0.47395 (15)	0.0349 (2)	0.0098 (3)	
O9	0.9282 (2)	0.57625 (14)	0.3373 (2)	0.0103 (3)	
O10	0.8758 (2)	0.75807 (15)	0.1281 (3)	0.0118 (3)	
O11	0.9976 (3)	0.21112 (16)	0.4905 (3)	0.0170 (4)	
O12	0.2081 (2)	0.74122 (15)	0.2372 (2)	0.0109 (3)	
O13	0.5208 (2)	0.80942 (16)	0.0672 (3)	0.0127 (3)	
O14	0.7740 (2)	0.13142 (15)	0.0878 (2)	0.0121 (3)	
O15	0.3966 (2)	0.96997 (15)	0.2470 (3)	0.0135 (3)	
H1	0.938 (5)	0.117 (3)	0.480 (5)	0.040*	

### Atomic displacement parameters (Å^2^)

**Table:**

	*U*^11^	*U*^22^	*U*^33^	*U*^12^	*U*^13^	*U*^23^
Li1	0.060 (5)	0.126 (8)	0.046 (4)	0.067 (6)	0.036 (4)	0.035 (5)
Ca1	0.0128 (2)	0.0071 (2)	0.0100 (2)	0.00122 (17)	0.00179 (16)	−0.00081 (16)
Ca2	0.0095 (2)	0.0128 (2)	0.0139 (2)	0.00159 (18)	−0.00014 (17)	−0.00341 (17)
Mn1	0.00686 (16)	0.00675 (16)	0.00782 (15)	0.00160 (12)	0.00084 (12)	−0.00022 (12)
Mn2	0.00669 (16)	0.00570 (15)	0.00900 (16)	0.00153 (12)	0.00000 (12)	−0.00028 (12)
Si1	0.0086 (3)	0.0066 (3)	0.0092 (3)	0.0012 (2)	−0.0004 (2)	−0.0006 (2)
Si2	0.0065 (3)	0.0060 (3)	0.0078 (3)	0.0006 (2)	0.0005 (2)	0.0000 (2)
Si3	0.0060 (3)	0.0054 (3)	0.0070 (3)	0.0011 (2)	0.0002 (2)	−0.0002 (2)
Si4	0.0076 (3)	0.0058 (3)	0.0088 (3)	0.0012 (2)	0.0005 (2)	−0.0002 (2)
Si5	0.0071 (3)	0.0072 (3)	0.0085 (3)	0.0021 (2)	0.0009 (2)	0.0000 (2)
O1	0.0164 (9)	0.0155 (9)	0.0103 (8)	−0.0036 (7)	0.0035 (7)	0.0019 (7)
O2	0.0107 (8)	0.0119 (8)	0.0118 (8)	0.0030 (7)	−0.0007 (6)	−0.0001 (6)
O3	0.0111 (8)	0.0059 (7)	0.0135 (8)	0.0020 (6)	−0.0011 (6)	−0.0011 (6)
O4	0.0096 (8)	0.0102 (8)	0.0118 (8)	0.0025 (6)	−0.0024 (6)	0.0001 (6)
O5	0.0112 (8)	0.0108 (8)	0.0109 (8)	0.0018 (7)	0.0014 (6)	−0.0026 (6)
O6	0.0085 (8)	0.0119 (8)	0.0104 (7)	0.0001 (6)	0.0017 (6)	0.0028 (6)
O7	0.0100 (8)	0.0103 (8)	0.0155 (8)	0.0044 (6)	0.0033 (6)	−0.0016 (6)
O8	0.0085 (7)	0.0114 (8)	0.0093 (7)	0.0024 (6)	−0.0003 (6)	0.0014 (6)
O9	0.0123 (8)	0.0052 (7)	0.0123 (8)	0.0010 (6)	−0.0007 (6)	−0.0002 (6)
O10	0.0107 (8)	0.0114 (8)	0.0142 (8)	0.0043 (7)	0.0003 (6)	0.0037 (6)
O11	0.0269 (10)	0.0094 (8)	0.0118 (8)	−0.0008 (8)	0.0078 (7)	−0.0032 (6)
O12	0.0078 (8)	0.0118 (8)	0.0126 (8)	0.0010 (6)	0.0019 (6)	0.0036 (6)
O13	0.0109 (8)	0.0127 (8)	0.0164 (8)	0.0053 (7)	0.0046 (7)	0.0018 (7)
O14	0.0138 (8)	0.0121 (8)	0.0100 (8)	0.0032 (7)	−0.0016 (6)	0.0029 (6)
O15	0.0128 (8)	0.0099 (8)	0.0171 (8)	0.0029 (7)	0.0006 (7)	−0.0036 (6)

### Geometric parameters (Å, °)

**Table:**

Li1—O14	1.956 (7)	Mn2—O14^vii^	2.1304 (18)
Li1—O3	2.004 (7)	Mn2—O11^vii^	2.1778 (18)
Li1—O1^i^	2.121 (8)	Mn2—O4	2.1912 (17)
Li1—O15^ii^	2.336 (11)	Mn2—O2	2.2180 (18)
Li1—O11	2.642 (10)	Mn2—O10^v^	2.2396 (18)
Li1—O6	2.869 (11)	Si1—O2	1.6068 (18)
Ca1—O1^iii^	2.2873 (19)	Si1—O1	1.6105 (19)
Ca1—O13	2.3074 (19)	Si1—O3	1.6346 (18)
Ca1—O14^iv^	2.3441 (18)	Si1—O15^ii^	1.6431 (19)
Ca1—O2^v^	2.3506 (18)	Si2—O5^iii^	1.5891 (18)
Ca1—O10	2.3534 (18)	Si2—O4	1.6119 (17)
Ca1—O2^vi^	2.4648 (18)	Si2—O3	1.6502 (17)
Ca2—O7^vii^	2.3119 (18)	Si2—O6	1.6690 (18)
Ca2—O8^v^	2.3429 (18)	Si3—O7	1.5856 (19)
Ca2—O5	2.3455 (18)	Si3—O8	1.6035 (17)
Ca2—O4	2.3688 (18)	Si3—O6	1.6428 (18)
Ca2—O6^iii^	2.4857 (18)	Si3—O9	1.6709 (18)
Ca2—O9^vii^	2.6230 (19)	Si4—O10	1.6033 (18)
Ca2—O12	2.6477 (18)	Si4—O11^viii^	1.6129 (19)
Mn1—O13	2.1268 (18)	Si4—O9	1.6374 (18)
Mn1—O5	2.1281 (17)	Si4—O12^ix^	1.6542 (19)
Mn1—O10	2.1952 (18)	Si5—O13	1.5900 (19)
Mn1—O8^v^	2.2010 (17)	Si5—O14^v^	1.6125 (17)
Mn1—O4^v^	2.2635 (18)	Si5—O15	1.6560 (18)
Mn1—O8	2.2941 (18)	Si5—O12	1.6695 (18)
Mn2—O7^vii^	2.0704 (17)		
			
O2—Si1—O1	113.72 (10)	O7—Si3—O9	102.31 (9)
O2—Si1—O3	114.40 (9)	O8—Si3—O9	111.55 (9)
O1—Si1—O3	107.47 (9)	O6—Si3—O9	104.52 (9)
O2—Si1—O15^ii^	110.10 (10)	O10—Si4—O11^viii^	112.69 (11)
O1—Si1—O15^ii^	107.53 (10)	O10—Si4—O9	113.18 (9)
O3—Si1—O15^ii^	102.87 (10)	O11^viii^—Si4—O9	107.23 (10)
O5^iii^—Si2—O4	115.99 (10)	O10—Si4—O12^ix^	109.06 (9)
O5^iii^—Si2—O3	114.15 (9)	O11^viii^—Si4—O12^ix^	109.23 (10)
O4—Si2—O3	108.16 (9)	O9—Si4—O12^ix^	105.14 (9)
O5^iii^—Si2—O6	103.34 (9)	O13—Si5—O14^v^	113.97 (10)
O4—Si2—O6	111.94 (9)	O13—Si5—O15	108.72 (10)
O3—Si2—O6	102.39 (9)	O14^v^—Si5—O15	106.07 (9)
O7—Si3—O8	116.88 (10)	O13—Si5—O12	110.06 (10)
O7—Si3—O6	111.46 (10)	O14^v^—Si5—O12	110.83 (9)
O8—Si3—O6	109.28 (9)	O15—Si5—O12	106.85 (9)

Symmetry codes: (i) −*x*+1, −*y*, −*z*+1; (ii) *x*, *y*−1, *z*; (iii) −*x*+1, −*y*+1, −*z*+1; (iv) *x*, *y*+1, *z*; (v) −*x*+1, −*y*+1, −*z*; (vi) *x*+1, *y*+1, *z*; (vii) *x*−1, *y*, *z*; (viii) −*x*+2, −*y*+1, −*z*+1; (ix) *x*+1, *y*, *z*.

### Hydrogen-bond geometry (Å, °)

**Table:**

*D*—H···*A*	*D*—H	H···*A*	*D*···*A*	*D*—H···*A*
O11—H1···O1^i^	1.07 (4)	1.44 (4)	2.462 (3)	157 (3)

Symmetry codes: (i) −*x*+1, −*y*, −*z*+1.
